# 4-({[6-(4-Chloro­benz­yl)-4-methyl-5-oxo-4,5-dihydro-1,2,4-triazin-3-yl]sulfan­yl}acetyl)-3-phenyl­sydnone

**DOI:** 10.1107/S1600536811007689

**Published:** 2011-03-09

**Authors:** Hoong-Kun Fun, Madhukar Hemamalini, Nithinchandra, Balakrishna Kalluraya

**Affiliations:** a X-ray Crystallography Unit, School of Physics, Universiti Sains Malaysia, 11800 USM, Penang, Malaysia; b Department of Studies in Chemistry, Mangalore University, Mangalagangotri, Mangalore 574 199, India

## Abstract

In the title syndone (1,2,3-oxadiazol-3-ylium-5-olate) compound, C_21_H_16_ClN_5_O_4_S, the dihedral angle between the benzene and oxadiazole rings is 55.62 (11)° and that between the triazine and the chloro-substituted phenyl rings is 82.45 (9)°. There is an intra­molecular C—H⋯S hydrogen bond, which generates an *S*(5) ring motif. In the crystal, inversion dimers linked by pairs of C—H⋯O hydrogen bonds generate *R*_2_^2^(20) loops. The dimers are connected by C—H⋯N and C—H⋯O hydrogen bonds.

## Related literature

For applications of sydnones, see: Rai *et al.* (2008 ▶); Jyothi *et al.* (2008 ▶).
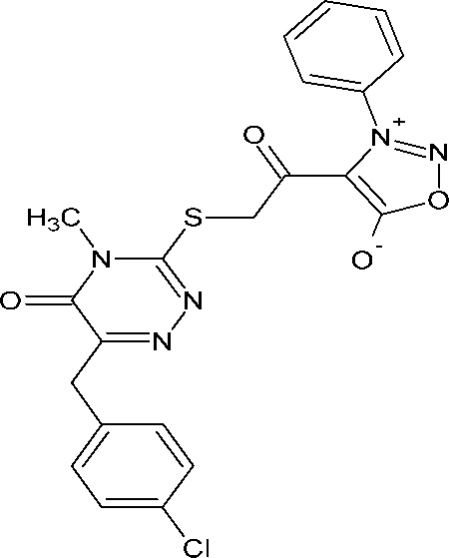


## Experimental

### 

#### Crystal data

C_21_H_16_ClN_5_O_4_S
*M* *_r_* = 469.90
Triclinic, 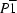

*a* = 6.4604 (1) Å
*b* = 10.1634 (2) Å
*c* = 16.9901 (4) Å
α = 105.264 (1)°
β = 92.103 (1)°
γ = 97.363 (1)°
*V* = 1064.44 (4) Å^3^
*Z* = 2
Mo *K*α radiation
μ = 0.32 mm^−1^
*T* = 296 K
0.62 × 0.39 × 0.13 mm

#### Data collection

Bruker SMART APEXII CCD area-detector diffractometer
Absorption correction: multi-scan (*SADABS*; Bruker, 2009) ▶ *T*_min_ = 0.828, *T*_max_ = 0.959
21841 measured reflections
6389 independent reflections
4781 reflections with *I* > 2σ(*I*)
*R*_int_ = 0.020

#### Refinement

*R*[*F* ^2^ > 2σ(*F* ^2^)] = 0.049
*wR*(*F* ^2^) = 0.154
*S* = 1.05
6389 reflections
290 parameters
H-atom parameters constrained
Δρ_max_ = 0.63 e Å^−3^
Δρ_min_ = −0.49 e Å^−3^

### 

Data collection: *APEX2* (Bruker, 2009 ▶); cell refinement: *SAINT* (Bruker, 2009 ▶); data reduction: *SAINT*; program(s) used to solve structure: *SHELXTL* (Sheldrick, 2008 ▶); program(s) used to refine structure: *SHELXTL*; molecular graphics: *SHELXTL*; software used to prepare material for publication: *SHELXTL* and *PLATON* (Spek, 2009 ▶).

## Supplementary Material

Crystal structure: contains datablocks global, I. DOI: 10.1107/S1600536811007689/hb5811sup1.cif

Structure factors: contains datablocks I. DOI: 10.1107/S1600536811007689/hb5811Isup2.hkl

Additional supplementary materials:  crystallographic information; 3D view; checkCIF report

## Figures and Tables

**Table 1:** Hydrogen-bond geometry (Å, °)

*D*—H⋯*A*	*D*—H	H⋯*A*	*D*⋯*A*	*D*—H⋯*A*
C7—H7*B*⋯O2^i^	0.97	2.52	3.429 (3)	157
C11—H11*A*⋯S1	0.96	2.12	2.7577 (17)	122
C11—H11*B*⋯N1^ii^	0.96	2.36	3.066 (2)	130
C11—H11*B*⋯N2^ii^	0.96	2.40	3.0304 (19)	123
C11—H11*C*⋯O1^iii^	0.96	2.07	3.021 (2)	169

Symmetry codes: (i) 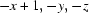; (ii) 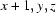; (iii) 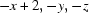.

**Fig. 1.**
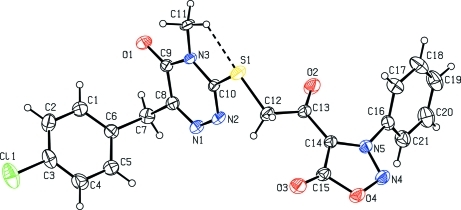
The asymmetric unit of the title compound, showing 30% probability displacement ellipsoids. The intramolecular hydrogen bond is shown as a dashed line.

**Fig. 2.**
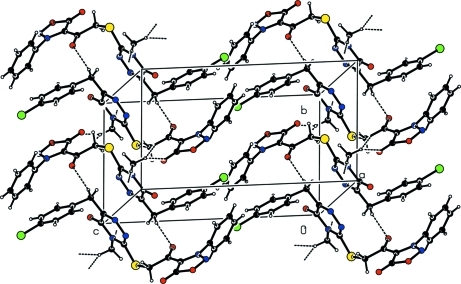
The crystal packing of the title compound (I).

## supplementary crystallographic information

### Comment

Sydnones (1,2,3-oxadiazol-3-ylium-5-olates) are mesoionic heterocyclic aromatic compounds. The study of sydnones still remains a field of interest because of their electronic structures and also because of the varied types of biological activities being reported (Rai *et al.*, 2008). Recently sydnone derivatives were found to exhibit promising anti-microbial properties (Jyothi *et al.*, 2008). Since their discovery, sydnones have shown diverse biological activities and it is thought that the meso-ionic nature of the sydnone ring promotes significant interactions with biological systems. Photochemical bromination of 3-aryl-4-acetylsydnone afforded 3-aryl-4 bromoacetylsydnones.  Condensation of 6-(4-chlorobenzyl)-4-methyl-3- sulfanyl-1,2,4-triazin-5(4*H*)-one with 3-aryl-4-bromoacetylsydnones yielded *S*-substituted triazinone derivatives (Jyothi *et al.*, 2008).

In the title compound (Fig. 1), the rings A (C16–C21), B (N4/N5/O4/C14–C15), C (N1/N2/N3/C8–C10) and D (C1–C6) are essentially planar. The dihedral angle between the best planes of the rings are A/B = 55.62 (11)°, A/C = 83.22 (10)°, A/D = 49.75 (11)°, B/C = 87.81 (9)°, B/D = 8.97 (10)° and C/D = 82.45 (9)°.

In the crystal (Fig. 2),  symmetry-related molecules are linked into centrosymmetic dimers via pairs of intermolecular C—H···O hydrogen bonds, generating an *R*^2^_2_(20) ring. Furthermore, these dimers are connected *via* C—H···N and C—H···O hydrogen bonds. There is an intramolecular C—H···S hydrogen bond, which generates an *S*(5) ring motif.

### Experimental

To a mixture of 4-bromoacetyl-3-phenylsydnone (0.01 mol) and 6-(4-chlorobenzyl)-4-methyl-3-sulfanyl-1,2,4-triazin-5(4*H*)-one (0.01 mol) in ethanol, a catalytic amount of anhydrous sodium acetate was added. The solution was stirred at room temperature for 2-3hours. The solid product separated was filtered and dried.  It was then recrystallized from ethanol. Colourless plates of (I) were obtained from 1:2 mixtures of DMF and ethanol by slow evaporation.

### Refinement

All H atoms were positioned geometrically [C–H = 0.93–0.97 Å] and were refined  using a riding model, with *U*_iso_(H) = 1.2 or 1.5 *U*_eq_(C).

### Crystal data

**Table:**

C_21_H_16_ClN_5_O_4_S	*Z* = 2
*M**_r_* = 469.90	*F*(000) = 484
Triclinic, *P*1	*D*_x_ = 1.466 Mg m^−^^3^
Hall symbol: -P 1	Mo *K*α radiation, λ = 0.71073 Å
*a* = 6.4604 (1) Å	Cell parameters from 9469 reflections
*b* = 10.1634 (2) Å	θ = 2.5–30.3°
*c* = 16.9901 (4) Å	µ = 0.32 mm^−^^1^
α = 105.264 (1)°	*T* = 296 K
β = 92.103 (1)°	Plate, colourless
γ = 97.363 (1)°	0.62 × 0.39 × 0.13 mm
*V* = 1064.44 (4) Å^3^	

### Data collection

**Table:**

Bruker SMART APEXII CCD area-detector diffractometer	6389 independent reflections
Radiation source: fine-focus sealed tube	4781 reflections with *I* > 2σ(*I*)
graphite	*R*_int_ = 0.020
φ and ω scans	θ_max_ = 30.4°, θ_min_ = 2.1°
Absorption correction: multi-scan (*SADABS*; Bruker, 2009)	*h* = −9→9
*T*_min_ = 0.828, *T*_max_ = 0.959	*k* = −14→14
21841 measured reflections	*l* = −24→23

### Refinement

**Table:**

Refinement on *F*^2^	Primary atom site location: structure-invariant direct methods
Least-squares matrix: full	Secondary atom site location: difference Fourier map
*R*[*F*^2^ > 2σ(*F*^2^)] = 0.049	Hydrogen site location: inferred from neighbouring sites
*wR*(*F*^2^) = 0.154	H-atom parameters constrained
*S* = 1.05	*w* = 1/[σ^2^(*F*_o_^2^) + (0.0757*P*)^2^ + 0.3213*P*] where *P* = (*F*_o_^2^ + 2*F*_c_^2^)/3
6389 reflections	(Δ/σ)_max_ < 0.001
290 parameters	Δρ_max_ = 0.63 e Å^−^^3^
0 restraints	Δρ_min_ = −0.49 e Å^−^^3^

### Special details

**Table:**

Geometry. All s.u.'s (except the s.u. in the dihedral angle between two l.s. planes) are estimated using the full covariance matrix. The cell s.u.'s are taken into account individually in the estimation of s.u.'s in distances, angles and torsion angles; correlations between s.u.'s in cell parameters are only used when they are defined by crystal symmetry. An approximate (isotropic) treatment of cell s.u.'s is used for estimating s.u.'s involving l.s. planes.
Refinement. Refinement of F^2^ against ALL reflections. The weighted R-factor wR and goodness of fit S are based on F^2^, conventional R-factors R are based on F, with F set to zero for negative F^2^. The threshold expression of F^2^ > 2σ(F^2^) is used only for calculating R-factors(gt) etc. and is not relevant to the choice of reflections for refinement. R-factors based on F^2^ are statistically about twice as large as those based on F, and R- factors based on ALL data will be even larger.

### Fractional atomic coordinates and isotropic or equivalent isotropic displacement parameters (Å^2^)

**Table:**

	*x*	*y*	*z*	*U*_iso_*/*U*_eq_	
Cl1	0.62801 (16)	0.17208 (7)	−0.44701 (4)	0.0927 (3)	
S1	0.74666 (7)	0.42674 (5)	0.10616 (3)	0.04942 (14)	
O1	0.8261 (2)	−0.00776 (14)	−0.09214 (9)	0.0536 (3)	
O2	0.4372 (2)	0.37342 (15)	0.22327 (10)	0.0631 (4)	
O3	0.2131 (2)	0.70857 (15)	0.14094 (9)	0.0569 (4)	
O4	−0.0096 (2)	0.68960 (14)	0.23895 (9)	0.0525 (3)	
N1	0.3637 (2)	0.14727 (17)	−0.05789 (10)	0.0455 (3)	
N2	0.4389 (2)	0.26204 (16)	0.00528 (9)	0.0432 (3)	
N3	0.76804 (19)	0.18731 (14)	0.00212 (8)	0.0357 (3)	
N4	−0.0422 (3)	0.61324 (17)	0.29404 (10)	0.0513 (4)	
N5	0.1021 (2)	0.53269 (14)	0.28321 (9)	0.0391 (3)	
C1	0.6273 (3)	−0.0406 (2)	−0.28017 (12)	0.0538 (5)	
H1A	0.7109	−0.0981	−0.2639	0.065*	
C2	0.6816 (4)	0.0139 (2)	−0.34463 (13)	0.0614 (5)	
H2A	0.8010	−0.0068	−0.3715	0.074*	
C3	0.5567 (4)	0.0987 (2)	−0.36822 (12)	0.0573 (5)	
C4	0.3787 (4)	0.1282 (2)	−0.33058 (13)	0.0625 (6)	
H4A	0.2938	0.1839	−0.3481	0.075*	
C5	0.3265 (4)	0.0743 (2)	−0.26612 (13)	0.0559 (5)	
H5A	0.2063	0.0950	−0.2398	0.067*	
C6	0.4501 (3)	−0.01022 (17)	−0.23998 (11)	0.0429 (4)	
C7	0.3994 (3)	−0.05628 (19)	−0.16429 (12)	0.0491 (4)	
H7A	0.2491	−0.0782	−0.1632	0.059*	
H7B	0.4621	−0.1381	−0.1645	0.059*	
C8	0.4848 (3)	0.05888 (18)	−0.09049 (10)	0.0391 (3)	
C9	0.7063 (3)	0.07191 (17)	−0.06296 (10)	0.0381 (3)	
C10	0.6336 (2)	0.27910 (17)	0.03115 (10)	0.0351 (3)	
C11	0.9776 (2)	0.22301 (18)	0.03403 (11)	0.0415 (4)	
H11A	0.9900	0.3032	0.0799	0.062*	
H11B	1.0642	0.2424	−0.0074	0.062*	
H11C	1.0218	0.1478	0.0515	0.062*	
C12	0.5200 (3)	0.51093 (19)	0.13034 (12)	0.0451 (4)	
H12A	0.5638	0.6097	0.1483	0.054*	
H12B	0.4277	0.4916	0.0811	0.054*	
C13	0.3992 (3)	0.46689 (17)	0.19597 (10)	0.0396 (3)	
C14	0.2332 (2)	0.54916 (16)	0.22475 (10)	0.0364 (3)	
C15	0.1633 (3)	0.65315 (17)	0.19282 (11)	0.0421 (4)	
C16	0.0970 (3)	0.44216 (18)	0.33652 (10)	0.0433 (4)	
C17	0.2718 (4)	0.4479 (2)	0.38688 (13)	0.0567 (5)	
H17A	0.3960	0.5035	0.3843	0.068*	
C18	0.2562 (5)	0.3678 (3)	0.44141 (15)	0.0719 (7)	
H18A	0.3719	0.3693	0.4760	0.086*	
C19	0.0713 (5)	0.2859 (3)	0.44518 (15)	0.0728 (7)	
H19A	0.0623	0.2345	0.4832	0.087*	
C20	−0.1000 (5)	0.2797 (3)	0.39297 (16)	0.0711 (6)	
H20A	−0.2230	0.2223	0.3947	0.085*	
C21	−0.0893 (3)	0.3590 (2)	0.33778 (13)	0.0568 (5)	
H21A	−0.2043	0.3564	0.3026	0.068*	

### Atomic displacement parameters (Å^2^)

**Table:**

	*U*^11^	*U*^22^	*U*^33^	*U*^12^	*U*^13^	*U*^23^
Cl1	0.1528 (8)	0.0725 (4)	0.0568 (4)	0.0023 (4)	0.0182 (4)	0.0294 (3)
S1	0.0344 (2)	0.0530 (3)	0.0574 (3)	0.01232 (18)	0.00630 (18)	0.0057 (2)
O1	0.0572 (8)	0.0531 (8)	0.0570 (8)	0.0312 (6)	0.0076 (6)	0.0144 (6)
O2	0.0748 (10)	0.0590 (9)	0.0762 (10)	0.0393 (8)	0.0273 (8)	0.0373 (8)
O3	0.0646 (9)	0.0527 (8)	0.0686 (9)	0.0205 (7)	0.0149 (7)	0.0356 (7)
O4	0.0538 (7)	0.0499 (7)	0.0648 (8)	0.0269 (6)	0.0156 (6)	0.0239 (6)
N1	0.0313 (7)	0.0565 (9)	0.0502 (8)	0.0091 (6)	0.0079 (6)	0.0148 (7)
N2	0.0291 (6)	0.0539 (8)	0.0484 (8)	0.0135 (6)	0.0088 (5)	0.0125 (7)
N3	0.0279 (6)	0.0444 (7)	0.0417 (7)	0.0138 (5)	0.0086 (5)	0.0188 (6)
N4	0.0519 (9)	0.0524 (9)	0.0575 (9)	0.0239 (7)	0.0160 (7)	0.0192 (7)
N5	0.0414 (7)	0.0383 (7)	0.0397 (7)	0.0121 (6)	0.0057 (5)	0.0105 (6)
C1	0.0592 (11)	0.0546 (11)	0.0531 (11)	0.0214 (9)	0.0073 (9)	0.0173 (9)
C2	0.0670 (13)	0.0644 (13)	0.0534 (12)	0.0163 (11)	0.0188 (10)	0.0119 (10)
C3	0.0891 (16)	0.0424 (10)	0.0387 (9)	0.0044 (10)	0.0046 (9)	0.0104 (8)
C4	0.0881 (16)	0.0538 (11)	0.0527 (11)	0.0292 (11)	0.0012 (11)	0.0184 (9)
C5	0.0625 (12)	0.0569 (11)	0.0535 (11)	0.0246 (10)	0.0068 (9)	0.0158 (9)
C6	0.0491 (9)	0.0370 (8)	0.0423 (9)	0.0083 (7)	0.0009 (7)	0.0094 (7)
C7	0.0521 (10)	0.0431 (9)	0.0537 (10)	0.0030 (8)	0.0048 (8)	0.0174 (8)
C8	0.0383 (8)	0.0416 (8)	0.0431 (8)	0.0066 (6)	0.0086 (6)	0.0204 (7)
C9	0.0416 (8)	0.0415 (8)	0.0406 (8)	0.0156 (7)	0.0108 (6)	0.0219 (7)
C10	0.0301 (7)	0.0432 (8)	0.0385 (8)	0.0127 (6)	0.0112 (5)	0.0178 (6)
C11	0.0232 (6)	0.0506 (9)	0.0503 (9)	0.0161 (6)	0.0015 (6)	0.0079 (7)
C12	0.0439 (9)	0.0445 (9)	0.0519 (10)	0.0170 (7)	0.0130 (7)	0.0155 (8)
C13	0.0411 (8)	0.0362 (8)	0.0438 (9)	0.0137 (6)	0.0056 (6)	0.0107 (7)
C14	0.0381 (8)	0.0344 (7)	0.0391 (8)	0.0107 (6)	0.0050 (6)	0.0115 (6)
C15	0.0439 (9)	0.0358 (8)	0.0501 (9)	0.0131 (7)	0.0062 (7)	0.0136 (7)
C16	0.0524 (10)	0.0429 (9)	0.0364 (8)	0.0105 (7)	0.0069 (7)	0.0117 (7)
C17	0.0613 (12)	0.0568 (11)	0.0534 (11)	0.0045 (9)	−0.0046 (9)	0.0204 (9)
C18	0.0925 (18)	0.0740 (15)	0.0542 (12)	0.0134 (14)	−0.0123 (12)	0.0280 (11)
C19	0.105 (2)	0.0678 (15)	0.0543 (13)	0.0086 (14)	0.0090 (12)	0.0322 (11)
C20	0.0845 (17)	0.0686 (15)	0.0654 (14)	−0.0010 (13)	0.0162 (12)	0.0313 (12)
C21	0.0565 (11)	0.0627 (12)	0.0536 (11)	0.0033 (9)	0.0070 (9)	0.0217 (10)

### Geometric parameters (Å, °)

**Table:**

Cl1—C3	1.744 (2)	C5—H5A	0.9300
S1—C10	1.7472 (18)	C6—C7	1.513 (3)
S1—C12	1.7960 (17)	C7—C8	1.503 (3)
O1—C9	1.2159 (19)	C7—H7A	0.9700
O2—C13	1.209 (2)	C7—H7B	0.9700
O3—C15	1.197 (2)	C8—C9	1.468 (2)
O4—N4	1.370 (2)	C11—H11A	0.9600
O4—C15	1.420 (2)	C11—H11B	0.9600
N1—C8	1.295 (2)	C11—H11C	0.9600
N1—N2	1.381 (2)	C12—C13	1.513 (2)
N2—C10	1.292 (2)	C12—H12A	0.9700
N3—C10	1.3655 (18)	C12—H12B	0.9700
N3—C9	1.386 (2)	C13—C14	1.463 (2)
N3—C11	1.4055 (19)	C14—C15	1.422 (2)
N4—N5	1.3046 (19)	C16—C17	1.377 (3)
N5—C14	1.358 (2)	C16—C21	1.383 (3)
N5—C16	1.449 (2)	C17—C18	1.383 (3)
C1—C6	1.381 (3)	C17—H17A	0.9300
C1—C2	1.388 (3)	C18—C19	1.379 (4)
C1—H1A	0.9300	C18—H18A	0.9300
C2—C3	1.375 (3)	C19—C20	1.377 (4)
C2—H2A	0.9300	C19—H19A	0.9300
C3—C4	1.365 (3)	C20—C21	1.386 (3)
C4—C5	1.382 (3)	C20—H20A	0.9300
C4—H4A	0.9300	C21—H21A	0.9300
C5—C6	1.387 (3)		
			
C10—S1—C12	100.06 (8)	N2—C10—S1	121.47 (12)
N4—O4—C15	110.86 (12)	N3—C10—S1	114.61 (11)
C8—N1—N2	120.99 (14)	N3—C11—H11A	109.5
C10—N2—N1	118.28 (14)	N3—C11—H11B	109.5
C10—N3—C9	121.17 (13)	H11A—C11—H11B	109.5
C10—N3—C11	117.25 (14)	N3—C11—H11C	109.5
C9—N3—C11	121.14 (13)	H11A—C11—H11C	109.5
N5—N4—O4	105.20 (13)	H11B—C11—H11C	109.5
N4—N5—C14	114.60 (14)	C13—C12—S1	113.61 (12)
N4—N5—C16	114.42 (14)	C13—C12—H12A	108.8
C14—N5—C16	130.97 (13)	S1—C12—H12A	108.8
C6—C1—C2	120.63 (18)	C13—C12—H12B	108.8
C6—C1—H1A	119.7	S1—C12—H12B	108.8
C2—C1—H1A	119.7	H12A—C12—H12B	107.7
C3—C2—C1	119.2 (2)	O2—C13—C14	122.62 (16)
C3—C2—H2A	120.4	O2—C13—C12	123.39 (15)
C1—C2—H2A	120.4	C14—C13—C12	113.98 (14)
C4—C3—C2	121.4 (2)	N5—C14—C15	105.91 (13)
C4—C3—Cl1	119.07 (17)	N5—C14—C13	126.38 (14)
C2—C3—Cl1	119.57 (19)	C15—C14—C13	127.49 (15)
C3—C4—C5	119.04 (19)	O3—C15—O4	120.05 (15)
C3—C4—H4A	120.5	O3—C15—C14	136.52 (17)
C5—C4—H4A	120.5	O4—C15—C14	103.43 (14)
C4—C5—C6	121.2 (2)	C17—C16—C21	122.79 (18)
C4—C5—H5A	119.4	C17—C16—N5	119.23 (17)
C6—C5—H5A	119.4	C21—C16—N5	117.87 (16)
C1—C6—C5	118.58 (18)	C16—C17—C18	117.6 (2)
C1—C6—C7	121.61 (17)	C16—C17—H17A	121.2
C5—C6—C7	119.59 (17)	C18—C17—H17A	121.2
C8—C7—C6	108.29 (14)	C19—C18—C17	120.9 (2)
C8—C7—H7A	110.0	C19—C18—H18A	119.5
C6—C7—H7A	110.0	C17—C18—H18A	119.5
C8—C7—H7B	110.0	C20—C19—C18	120.3 (2)
C6—C7—H7B	110.0	C20—C19—H19A	119.8
H7A—C7—H7B	108.4	C18—C19—H19A	119.8
N1—C8—C9	123.14 (16)	C19—C20—C21	120.1 (2)
N1—C8—C7	118.42 (16)	C19—C20—H20A	120.0
C9—C8—C7	118.25 (15)	C21—C20—H20A	120.0
O1—C9—N3	122.23 (16)	C16—C21—C20	118.2 (2)
O1—C9—C8	125.41 (17)	C16—C21—H21A	120.9
N3—C9—C8	112.36 (13)	C20—C21—H21A	120.9
N2—C10—N3	123.91 (16)		
			
C8—N1—N2—C10	0.0 (2)	C11—N3—C10—S1	−1.28 (19)
C15—O4—N4—N5	−0.1 (2)	C12—S1—C10—N2	4.33 (16)
O4—N4—N5—C14	0.5 (2)	C12—S1—C10—N3	−176.91 (12)
O4—N4—N5—C16	179.66 (14)	C10—S1—C12—C13	87.66 (14)
C6—C1—C2—C3	0.0 (3)	S1—C12—C13—O2	−7.7 (2)
C1—C2—C3—C4	−1.3 (3)	S1—C12—C13—C14	171.37 (13)
C1—C2—C3—Cl1	177.98 (17)	N4—N5—C14—C15	−0.7 (2)
C2—C3—C4—C5	1.7 (3)	C16—N5—C14—C15	−179.67 (17)
Cl1—C3—C4—C5	−177.58 (17)	N4—N5—C14—C13	−175.56 (16)
C3—C4—C5—C6	−0.8 (3)	C16—N5—C14—C13	5.5 (3)
C2—C1—C6—C5	0.8 (3)	O2—C13—C14—N5	−0.6 (3)
C2—C1—C6—C7	−173.70 (19)	C12—C13—C14—N5	−179.66 (16)
C4—C5—C6—C1	−0.4 (3)	O2—C13—C14—C15	−174.35 (19)
C4—C5—C6—C7	174.22 (19)	C12—C13—C14—C15	6.6 (3)
C1—C6—C7—C8	94.1 (2)	N4—O4—C15—O3	179.13 (17)
C5—C6—C7—C8	−80.3 (2)	N4—O4—C15—C14	−0.28 (19)
N2—N1—C8—C9	0.8 (3)	N5—C14—C15—O3	−178.7 (2)
N2—N1—C8—C7	−174.08 (15)	C13—C14—C15—O3	−3.9 (4)
C6—C7—C8—N1	91.90 (19)	N5—C14—C15—O4	0.54 (18)
C6—C7—C8—C9	−83.27 (19)	C13—C14—C15—O4	175.34 (16)
C10—N3—C9—O1	176.61 (15)	N4—N5—C16—C17	−122.28 (19)
C11—N3—C9—O1	4.5 (2)	C14—N5—C16—C17	56.7 (3)
C10—N3—C9—C8	−3.8 (2)	N4—N5—C16—C21	54.0 (2)
C11—N3—C9—C8	−175.91 (14)	C14—N5—C16—C21	−127.0 (2)
N1—C8—C9—O1	−179.33 (17)	C21—C16—C17—C18	−1.0 (3)
C7—C8—C9—O1	−4.4 (2)	N5—C16—C17—C18	175.10 (19)
N1—C8—C9—N3	1.0 (2)	C16—C17—C18—C19	−0.3 (4)
C7—C8—C9—N3	175.98 (14)	C17—C18—C19—C20	1.7 (4)
N1—N2—C10—N3	−2.9 (2)	C18—C19—C20—C21	−1.8 (4)
N1—N2—C10—S1	175.76 (12)	C17—C16—C21—C20	0.8 (3)
C9—N3—C10—N2	5.0 (2)	N5—C16—C21—C20	−175.31 (19)
C11—N3—C10—N2	177.45 (16)	C19—C20—C21—C16	0.6 (4)
C9—N3—C10—S1	−173.73 (11)		

### Hydrogen-bond geometry (Å, °)

**Table:**

*D*—H···*A*	*D*—H	H···*A*	*D*···*A*	*D*—H···*A*
C7—H7B···O2^i^	0.97	2.52	3.429 (3)	157
C11—H11A···S1	0.96	2.12	2.7577 (17)	122
C11—H11B···N1^ii^	0.96	2.36	3.066 (2)	130
C11—H11B···N2^ii^	0.96	2.40	3.0304 (19)	123
C11—H11C···O1^iii^	0.96	2.07	3.021 (2)	169

Symmetry codes: (i) −*x*+1, −*y*, −*z*; (ii) *x*+1, *y*, *z*; (iii) −*x*+2, −*y*, −*z*.
